# Estimation of Wave Characteristics Based on Global Navigation Satellite System Data Installed on Board Sailboats

**DOI:** 10.3390/s19102295

**Published:** 2019-05-17

**Authors:** Paolo De Girolamo, Mattia Crespi, Alessandro Romano, Augusto Mazzoni, Marcello Di Risio, Davide Pasquali, Giorgio Bellotti, Myrta Castellino, Paolo Sammarco

**Affiliations:** 1 “Sapienza”, University of Rome, DICEA, Via Eudossiana, 18, 00184 Roma, Italy; paolo.degirolamo@uniroma1.it (P.D.G.); mattia.crespi@uniroma1.it (M.C.); alessandro.romano@uniroma1.it (A.R.); augusto.mazzoni@uniroma1.it (A.M.); myrta.castellino@uniroma1.it (M.C.); 2 Department of Civil, Construction-Architectural and Environmental Engineering (DICEAA)—Environmental and Maritime Hydraulic Laboratory (LIam), University of L’Aquila, 67100 L’Aquila, Italy; davide.pasquali@univaq.it; 3 Engineering Department, Roma Tre University, Via V. Volterra, 62, 00146 Roma, Italy; giorgio.bellotti@uniroma3.it; 4 Department of Civil Engineering and Computer Science , University of Rome “Tor Vergata”, DICII, Via del Politecnico, 1, 00133 Roma, Italy; sammarco@ing.uniroma2.it

**Keywords:** GPS data analysis, off-shore wave climate, sailboat, ship motions, wave characteristics

## Abstract

This paper illustrates a methodology to get a reliable estimation of the local wave properties, based on the reconstruction of the motion of a moving sailboat by means of GNSS receivers installed on board and an original kinematic positioning approach. The wave parameters reconstruction may be used for many useful practical purposes, e.g., to improve of autopilots, for real-time control systems of ships, to analyze and improve the performance of race sailboats, and to estimate the local properties of the waves. A Class 40 oceanic vessel (ECO40) left from the port of “Riva di Traiano” located close to Rome (Italy) on 19 October 2014 to perform a non-stop sailing alone around the world in energy and food self-sufficiency. The proposed system was installed on ECO40 and the proposed method was applied to estimate the wave properties during a storm in the Western Mediterranean Sea. The results compared against two sets of hindcast data and wave buoy records demonstrated the reliability of the method.

## 1. Introduction

Estimation of ship motions and wave properties from a traveling ship is of utmost importance for many ocean engineering applications [1], for example: (i) to improve the performance of autopilots and control systems of ships [2] or in general of floating structures [3]; (ii) to provide real-time wave data for Global Forecasting Systems (GFS) numerical models and for the calibration/validation of wave forecasting/hindcasting models (e.g., [4]), etc.; and (iii) to analyze the performance of a race sailboat, i.e., to derive actual velocity polar curves in the presence of waves and to correct wind measurements carried out onboard. As far as the estimation of the wave properties is concerned, a possible approach is to derive the parameters of interest directly on the basis of the ship motion, taking into account the dynamic ship frequency response function (e.g., [1,5,6,7]).

In the last years, the measurement of directional wave spectra on the basis of moving ships kinematic has been extensively studied (e.g., [8,9]). Some approximated solutions of the main theoretical problem arising from the transformation of encounter frequencies measured onboard into the true wave frequencies have been derived. This problem is known as the “triple-valued function problem”, which is due to the non-linear Doppler shift effect induced by frequency dispersive waves (e.g., [10,11]). Approximated solutions have been obtained by using both parametric methods, which assume the shape of the wave spectrum, and non-parametric (stochastic) methods, where the spectrum shape is not prescribed a priori [1,11,12].

Only in the last ten years, the GNSS technology has been applied on floating buoys for measuring directional wave spectra [13,14]. The advantages, as well as the disadvantages, of using GNSS buoys were pointed out by Herbers et al. [14]. On the other hand, onboard measurements of ship-motion is normally carried out by means of accelerometers used for standard pitch/roll directional wave buoys [10,11,15,16,17].

This paper describes the estimation of ship motion using GPS receivers, installed on a traveling sailboat for the evaluation of the local wave properties. It has to be stressed that the use of GNSS on an offshore traveling sailboat is challenging. Indeed, the distance of the moving GNSS receivers installed onboard (i.e., rover receivers) from reference static receivers of known coordinates (i.e., base stations) should not exceed few tens of kilometers. Indeed, Herbers et al. [14] used off-the-shelf GPS receivers able to provide positions with accuracy limited to a few meters, mentioning that sub-meter accuracy is achievable with post-processing methodology only. In this work, we propose to adopt the original methodology Kin-Vadase, presented and widely validated by Branzanti et al. [18], able to provide accuracies at decimeter level and at few millimeters/second for positions and velocities directly on-board, respectively (no need of external data, such as differential positioning or precise point positioning) and in real time. With respect to the work of De Girolamo et al. [19], this paper deals with a more detailed description of the methods and extends the results.

The paper is structured as follows. Section 2 illustrates the methodology to analyze the GNSS data and to use them in order to reconstruct the boat motion and, then, the wave parameters. Section 3 illustrates the real scale ECO40 sailboat used to test the proposed methodology during a non-stop sailing alone around the world. Section 4 discusses the results and demonstrates the reliability of the proposed method by comparing the estimated wave parameters against measured and hindcast data. Concluding remarks close the paper.

## 2. Methods

During offshore oceanic sailing, GPS measurements cannot be corrected using on land reference receivers, or a GNSS (Global Navigation Satellite System) network, since the distance between rovers and reference station should not be larger than few tens of kilometers. To solve this problem, in the present work, two GPS data processing techniques have been applied, by using a novel approach. The processed data provided by the GPS receivers have been used to compute the boat motion and to estimate the waves properties faced by the sailboat during its navigation around the world. This sections aims at detailing the proposed methods.

### 2.1. Analysis of Gnss Data

The GPS raw code and phase observations on both L1 and L2 frequencies were acquired by each GPS antenna/receiver system (in the following referred to as “receiver”) with a sampling rate of 2 Hz. The raw observations were stored on a flash-card by each receiver. The data analysis was carried out in post-processing after the recovery of the flash-cards. The analysis carried out in the post processing described in the following, however, can be carried out in real-time onboard of the sailboat. The post-processing was carried out by employing two different techniques: the Variometric approach and the Moving Base Kinematic.

The Variometric Approach for Displacements Analysis Standalone Engine (VADASE) is an innovative GPS data processing approach proposed in the recent past [20,21]. The approach is based on timing single differences of carrier phase observations continuously collected using a standalone GPS receiver and on standard GPS broadcast products (orbits and clocks) that are available in real-time. Therefore, one receiver works in standalone mode and the epoch-by-epoch displacements (equivalent to velocities) are estimated. In this work, a kinematic extension of the Variometric approach (Kin-VADASE) developed specifically for the navigation field was used.

A second approach was used. Indeed, the most widely used technique in GPS kinematic positioning is based on the using of two devices: the rover moving receiver and a reference static receiver of known coordinates. To obtain high accuracy results, the distance between the rover and the reference receiver should not exceed few tens of kilometers. Thus, it is almost impossible to apply this technique in off-shore navigation, due to the lack of close reference receivers availability. Nevertheless, in this work, we present an innovative application of differential kinematic positioning applied to a reference moving (hereinafter referred to as Moving Base Kinematic, MBK). With respect to this reference moving receiver, it was possible to estimate, epoch by epoch, the positions of another receiver. This technique does not allow defining the absolute position of the receiver but only the relative one, which is very accurate, since the distance between each couple of receivers is known. The results of the GPS data post processing may then be used to reconstruct the six Degrees of Freedom (hereinafter referred to as 6DOFs) boat motion and to estimate the wave parameters encountered by sailing boats (i.e., significant wave height and mean wave direction). Actually, only heave, roll and pitch motions are described as they are needed to estimate the waves parameters.

### 2.2. Boat Motion Reconstruction

This subsection describes the method used to reconstruct the 6DOFs boat motion by using the two approaches described in Section 2.1. The two approaches have been used in a complementary way. In the following, the methods used to compute each degree of freedom of the boat from GPS measurements needed to estimate the wave parameters are described. The boat 6DOFs are referred in the following to the center of the triangle defined by the three GPS positions and not to the center of gravity of the boat. However, a simple translation may be applied to the results in order to express the motion of the boat with respect to its center of gravity, as usual. It has to be stressed, however, that this work aims at estimating the wave parameters and that the 6DOF boat motion is used to reach the goal.

#### 2.2.1. Heave

To compute the boat heave, it is necessary to use a technique able to provide the absolute vertical position of the boat. Nevertheless, the up-velocity component provided by Kin-VADASE method is subjected to bias, mainly related to the number of visible satellites and to their position with respect to the GPS antennas location. A sensitivity analysis was performed by using different numerical techniques (i.e., first order, Simpson’s rule, etc.) to minimize the integration error. It is worth noting that heave motion should exhibit a mean value very close to zero. Nevertheless, whatever the used numerical technique, the bias on the velocities gives rise to a vertical movement of the antenna due to low frequency drift related to the numerical integration. To eliminate the bias, a high pass filter was used. The high pass filter was applied to the vertical movement time history obtained by integrating over time the up-velocity component provided by a GPS antenna. The cut-off frequency was chosen large enough to avoid the low frequency drift related to the numerical integration, but small enough to analyze both the sea and the swell wave features.

#### 2.2.2. Roll

For the computation of the boat roll motion, it is not necessary to use a technique able to provide the absolute position of the boat, as roll may be obtained by means of the relative vertical differences between two points located along an axis orthogonal to the main longitudinal axis of the boat.

The roll angle is not characterized by a zero mean value mainly because of two reasons. The first one is related to the wind acting on the sail and on the boat hull, which causes the heeling of the sailboat. It is a function of the wind speed and of the sails trim and configuration. The second one is due to the movable weight (i.e., ballast, not used sails, spare equipment, etc.), which are moved by the crew in order to reduce the heeling induced by the wind action. The heeling angle may be obtained by calculating the mean total roll angle value over a time longer than the waves encountered periods.

#### 2.2.3. Pitch

The computation of the pitch motion, as for the roll, can be carried out by using the moving base kinematic (MBK) technique. For this parameter, two GPS receivers located along the main longitudinal axis of the sailboat should be used. Nevertheless, other antennas configurations could be used (see Section 3). In this case, it has to be stressed that the signals may be affected by the influence of the roll motion. To get the estimation of the pitch signal but the roll component, a mean signal (i.e., the actual pitch) can be computed (see Section 4).

### 2.3. Wave Parameters Estimation

The synthetic parameters of the waves faced by sailboats can be derived from the heave, roll and pitch motions. First, the transfer function between the boat and the sea surface movements has to be evaluated and applied to the time series estimated on the basis of GNSS data. Then, the duration of the time window to be analyzed needs to be defined. Herein, time windows of 30 min were considered. Indeed, this duration, which is typical for standard analysis of the free surface elevation signals as measured from conventional wave buoys, is considered to be long enough to properly describe, in a statistical sense, the considered sea state and short enough for considering the wave features to be stationary [22]. On the one hand, the significant wave height may be directly inferred from the heave motion by performing standard time and frequency domain analyses. On the other hand, the directional spectrum may be estimated by using the Direct Fourier Transform Method (i.e., by means of the DIWASP package, e.g., [23]) applied to the heave, pitch and roll signals without taking into account the Doppler shift effect due to the sailboat traveling. It has to be stressed that the influence of the waves upon the roll angle may be obtained by subtracting the heeling angle from the total measured roll angle, obtaining in this way a signal characterized by a mean value which is equal to zero.

## 3. The Experimental Sailboat ECO40

The considered sailboat is a small oceanic race boat length overall of 12.0 m) characterized by a very light displacement. She is a Class 40 oceanic vessel named ECO40, which left from the port of “Riva di Traiano” located close to Rome (Italy) on 19 October 2014 to perform a non-stop sailing alone around the world in energy and food self-sufficiency. The boat route goes through the Gibraltar Strait, then descends the Atlantic Ocean and sailing around the Antarctic, at a mean latitude of 50° S, from west to east, rounding the famous capes of the world: Cape of Good Hope, Cape Leeuwin and Cape Horn. Finally, it ascends the Atlantic Ocean, passing again the Strait of Gibraltar and coming back to homeport [24,25].

The boat was equipped with three high precision GPS receivers (rovers), provided by Leica Geosystem, for measuring the movements of the boat. An ad hoc survey was performed just after their installation in order to know the positions of the GPS antennas needed to infer the three-dimensional position and attitude of the boat as a rigid body.

**Figure 1:**
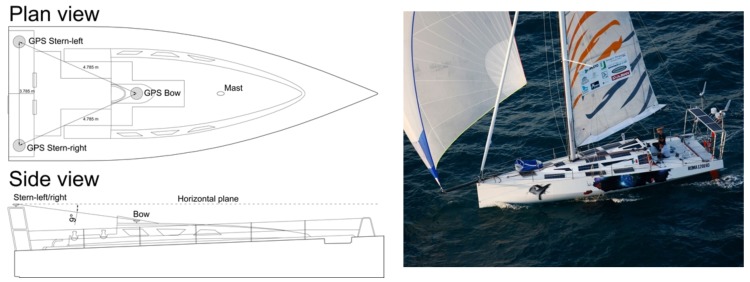
Plan view of the sailboat ECO40 and of the three GPS antennas (**Top Left**) and side view of the sailboat (**Bottom Left**); and (**Right**) a picture of the sailboat ECO40 during a test.

The three geodetic class GPS antenna/receiver systems are able to acquire code and phase observations on both L1 and L2 frequencies. The GPS antennas were installed on board ECO40, as shown in Figure 1. Two antennas were mounted on the stern roll bar (they are indicated as GPS Stern-right and GPS Stern-left in the figure) and one antenna (GPS Bow) was installed along the main axis of the boat, close to the entrance and protected by a small fiberglass structure. The positions of the three GPS antenna were at the vertices of an isosceles triangle, as described in the Figure 1. The proposed method was applied to three couples of receivers deployed on board (Figure 1): GPS Bow–GPS Stern-right; GPS Bow–GPS Stern-left; and GPS Stern-right–GPS Stern-left.

## 4. Results

**Figure 2:**
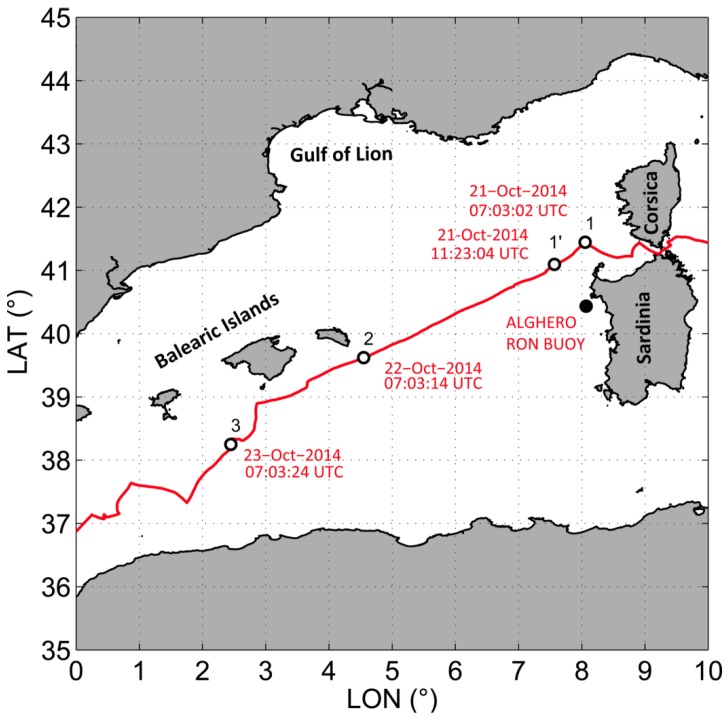
The route in the western Mediterranean Sea followed by ECO40. The empty dots refer to four points of interest along the route and the black dot identifies the position of the Alghero wave buoy.

When ECO40 left from the Italian Port Riva di Traiano (Italy) directed to Gibraltar Strait (on 19 October 2014), the weather forecasts suggested that, within the next 24/48 h, the first seasonal front of cold air was expected to induce Mistral winds with speed exceeding 40 knots, blowing from the Gulf of Lion [24]. ECO40 was able to reach the Asinara Island and to follow the route towards the Balearic Islands before the arrival of the main storm: the sailboat faced the storm running on the quarter. The route between the Asinara Island and the Balearic Islands is represented in Figure 2. The figure also provides information on the travel times.

This section aims at illustrating and discussing the results obtained by means of the proposed methodology described in Section 2.

**Figure 3:**
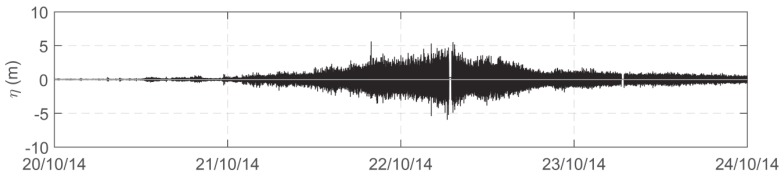
Example of the boat heave time series over a time window of four days, obtained by calculating the instant mean values of the three filtered heave time series as obtained by each GPS antenna.

The boat heave time series was obtained by computing the instantaneous mean values of the three filtered heave time series, as obtained by each of the three GPS antennas. Figure 3 shows an example of the calculated boat heave signal for a time window of four days.

**Figure 4:**
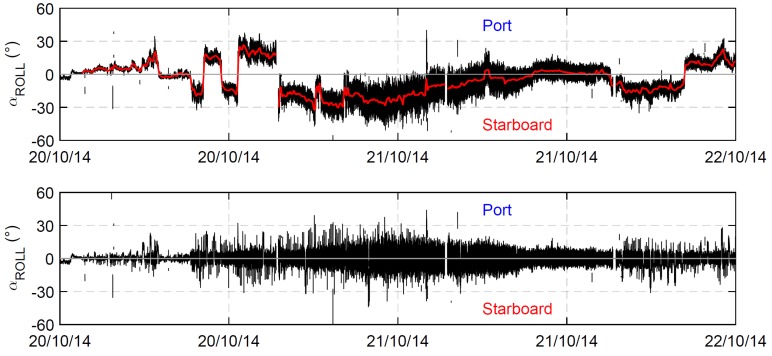
(**Top**) Example of the total measured roll angle time series (black line) over a time window of four4 days. The red line refers to the heeling angle calculated by averaging over 10 min. (**Bottom**) The roll angle obtained by subtracting the heeling angle to the total measured roll angle.

The roll motion angle was computed by using the moving base kinematic (MBK) applied to the relative Up position of the couple of Stern-GPSs. Figure 4 shows the roll angle during four days of navigation of ECO40. As expected, the analysis revealed that the measured roll angle was not characterized by a zero mean value. The red line in Figure 4 (top) shows the heeling angle computed over a time of 10 min. It is possible to identify the route changes of the boat which take place when the heeling angle changes from positive (port) to negative values (starboard) or vice versa. The influence of the waves upon the roll angle obtained by subtracting the heeling angle to the total measured roll angle is also shown in Figure 4 (bottom).

**Figure 5:**
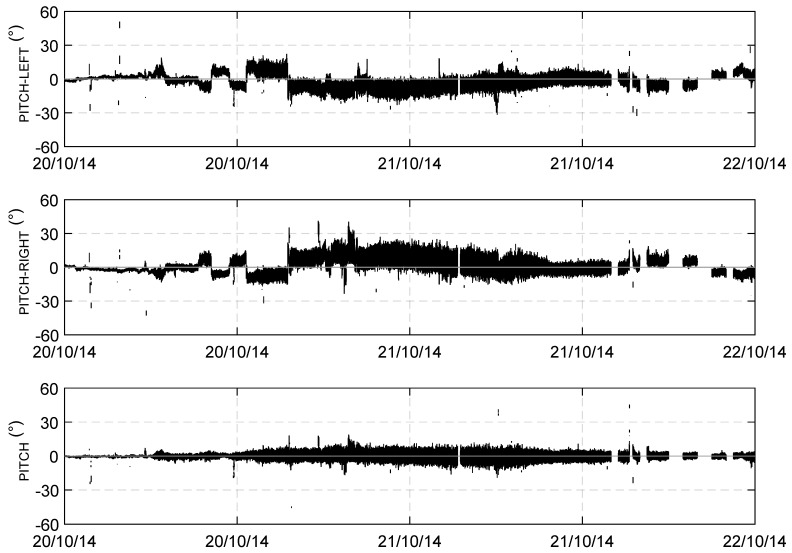
Time series of the angle obtained by using the bow–stern-left GPS couple (**Top**); and the same quantity as obtained by using the bow–stern-right GPS couple (**Middle**) over a time window of four days. (**Bottom**) Time series of the mean signal (pitch) obtained from the two couples bow–stern right and the bow–stern left over a time window of four days.

As far as the pith motion is considered, it has to be observed that two GPS receivers located along the main longitudinal axis of the sailboat were not available and therefore a direct estimation of the pitch could not be obtained. Two couples of GPS receivers were therefore considered: the bow–stern-right and the bow–stern-left. Figure 5 shows an example of the 2 Hz time series of the pitch angle obtained directly from the relative up position of each couple of GPS measurements. The time series were evaluated with respect to the local horizontal plane. Figure 5 (top) shows the time series of the pitch angle obtained by using the bow–stern-left GPS couple, while Figure 5 (middle) represents the same quantity as obtained by using the bow–stern-right GPS couple. The figure shows that both signals were contaminated by a component of the roll motion because the two axes passing for each couple of GPS (bow-stern right and bow-stern left) were not parallel to the main boat axis. Furthermore, the comparison of Figure 5 (top and middle) shows that the two signals are in phase opposition because of the changing of the reference GPS. To obtain the pitch signal depurated by the roll component, a mean signal obtained from the two couple bow–stern right and the bow–stern left was computed (Figure 5, bottom).

The features of the waves faced by the sailboat during the Gulf of Lion event were derived from the sailboat heave, roll and pitch motions. Since ECO40 is an ultra-light displacement boat (she has full loaded displacement of about 4700 kg, a length of about 12.0 m and a maximum breadth of 4.5 m), it was assumed that the sailboat follows the sea surface, thus assuming a unitary transfer function between the boat and sea surface movements (i.e., the response amplitude operator is the unit matrix).

**Figure 6:**
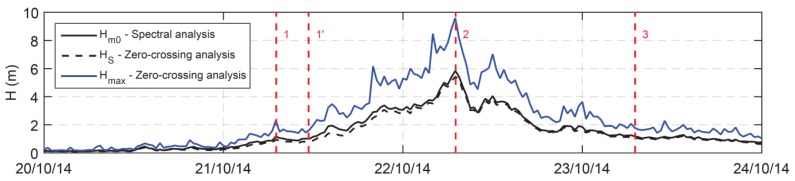
Significant wave height obtained from the heave by using zero crossing analysis (black dashed line) and frequency spectrum analysis (black line). The maximum wave height is reported as well (blue line). The vertical red dashed lines refer to the four points of interest along the route (as shown in Figure 2).

Figure 6 shows the significant wave height time series of the storm obtained from the heave signal by using both the zero-crossing analysis (H_1/3_) and the frequency spectrum analysis (H_*m*0_) carried out in the encountered frequency domain. The maximum zero crossing wave height (H_*max*_) is also represented. The maximum values obtained during the storm for H_1/3_ and for H_*m*0_ were, respectively, 5.84 m and 5.42 m, while H_*max*_ was of 9.6 m. The vertical dashed lines refer to four points of interest along the route (as shown in Figure 2).

**Figure 7:**
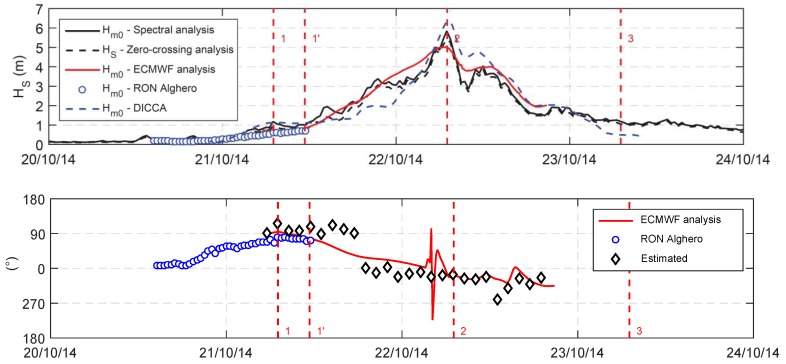
(**Top**) Comparison between the significant wave height measured onboard and the available wave data; and (**Bottom**) comparison between the mean spectral wave direction as estimated by means of the proposed method and the same quantity as obtained by the ECMWF data (red line) and Alghero wave buoy data (blue dots). The red dashed lines refer to four points of interest along the route (as shown in Figure 2).

To assess the accuracy of the estimated wave parameters, Figure 7 shows the comparison between the significant wave height and mean wave direction as: (i) measured by the Alghero RON wave buoy; (ii) estimated by the ECMWF analysis; and (iii) estimated by DICCA for a grid point close to the buoy position. It has to be stressed that this study aimed at estimating the wave parameters based on GNSS data analysis (see Section 2). Therefore, only the synthetic wave parameters were compared to the results of other research works, being the original methodology Kin-VADASE widely validated (see Section 2, [18]) even if applied to a different environment.The Alghero wave buoy belongs to the “RON-Rete Ondametrica Nazionale” (Italian Wave Measurements Network [26]), managed by ISPRA up to 2014 and now dismissed after about 23 years; the buoy was located offshore the northwest coast of Sardinia and it was exposed to the waves generated by the storm at hand. The six-hourly hindcast wave data provided in analysis by ECMWF (European Centre for Medium-Range Weather Forecasts) and the the hourly wave hindcast data provided by DICCA (Department of Civil, Chemical and Environmental Engineering, University of Genoa, Italy), covering 36 (1979–2014) years over the Mediterranean Sea [27] were also considered. were interpolated in time and in space by means of bi-linear technique. Since the buoy was operating at a fixed position, the comparison between the buoy wave data and the ones measured onboard was performed just for the time window in which the two measurements can be reasonably compared. The analysis revealed that the synthetic parameters reconstructed on the basis of sailboat motion was in close agreement with the DICCA data. On the other hand, the ECMWF data seemed to underestimate the storm peak, as already outlined by previous work e.g., [4], likely due to the time and spatial resolution of the hindcast data. As far as the mean wave direction is concerned, the accuracy of the proposed method was reasonable.

## 5. Concluding Remarks

A method to reconstruct the movements of a sailboat in order to get a reliable estimate of wave parameters, using GPS receivers, is presented. It is based on the combined approach of the Kin-VADASE and Moving Base Kinematic techniques. The proposed method was applied to the records collected onboard the ECO40 sailboat during a storm in the Western Mediterranean Sea. Three GPS antennas were deployed on the sailboat. Their positions were measured by means of a survey performed when they were installed. Therefore, the three-dimensional locations of the GPS antennas suffice to infer the three-dimensional position and attitude of the boat as a rigid body, hence to reconstruct wave parameters. A fourth GPS antenna could be considered to improve the reliability of the estimation of the position and attitude of the boat.

Wave properties as derived on the basis of the estimated boat movements were compared to available wave data. It appears that the wave properties were calculated with a high degree of accuracy. Note that the main aim of this work was the estimation of wave parameters, hence the GNSS data analysis was not directly validated. Of course, the good agreement between estimated and available data may be viewed as an indirect validation of GNSS data analysis as well.

In general, the techniques proposed in this paper appear to be suitable to estimate the 6DOFs movements of sailboats navigating offshore, where onshore reference receivers or GNSS network cannot be used to correct GPS errors. The motion of the boat can provide quantitative information for many important practical applications, such as the improvement of autopilots, the analysis and the optimization of ship performances, and the estimation of local wave properties. Furthermore, it can be used to correct wind measurements carried out onboard sailboats.
